# A Secondary Analysis of Longitudinal Pilot‐Study Data Investigating the Associations Between Health‐Related Quality of Life and Executive Functions in Remitted Major Depressive Disorder and Developments Two‐Years Following Cognitive Training

**DOI:** 10.1111/sjop.70060

**Published:** 2025-12-14

**Authors:** Eivind Haga Ronold, Silje Klundelien Storfossen, Marta Vikingstad Vignes, Åsa Hammar

**Affiliations:** ^1^ Department of Biological and Medical Psychology, Faculty of Psychology University of Bergen Bergen Norway; ^2^ Department of Clinical Sciences Lund, Psychiatry, Faculty of Medicine Lund University Lund Sweden; ^3^ Department of Psychiatry Skåne University Hospital Lund Sweden

**Keywords:** cognitive deficits, computerized working memory training, executive function, health‐related quality of life, longitudinal study, major depressive disorder, remission

## Abstract

Major depressive disorder (MDD) is associated with reduced quality of life and relapse risk. However, few studies have investigated how quality of life is associated with cognitive deficits following MDD and is affected by cognitive training. This study investigated the long‐term effects of computer‐based working memory training (CWMT) on health‐related quality of life (HRQL) in remitted MDD, and the association between executive functions (EF) and HRQL. Twenty‐nine remitted participants (*M* age 36.21, SD = 10.8) were included in a pre‐post pilot study of CWMT with 1‐ and 2‐year follow‐up. Twenty participants completed 5 weeks of CWMT, 12 participants were included at the 1‐year follow‐up, and 10 participants returned for the 2‐year follow‐up. The 36‐item Short‐Form Health Survey was used to assess HRQL. Associations between subjective and objective cognitive EF and HRQL were measured by the Behavior Rating Inventory of Executive Function for Adults (BRIEF‐A) and a neuropsychological test battery of EF. Significant moderate improvements were found in aspects of HRQL after 2 years (*d* = 0.66). There were negative correlations between HRQL and BRIEF‐A pre‐intervention (*r* = 0.47–0.65). However, the study did not find significant associations between improved EF and improved HRQL. Preliminary results indicate long‐term improvements in HRQL following CWMT. Subjective EF deficits were associated with poorer HRQL. However, due to limitations including small sample size and multiple statistical comparisons, larger controlled studies are needed to investigate and replicate the potential effects of CWMT on HRQL.

## Key Points

- Associations were found between self‐reported but not objectively measured executive functions and health‐related quality of life in remitted depression.
- Health‐related quality of life improved two years following Computer‐based Working Memory training.
- This could indicate that the benefits from Computer‐based Working Memory training may take time to manifest in everyday functioning.
- Future cognitive remediation studies should include long‐term follow‐up to assess transfer effects to daily functioning.

## 1 Introduction

An estimated 4.4% of the world's population is suffering from major depressive disorder (MDD), which is ranked as the largest contributor to disability and cause of burden globally (Ferrari et al. 2013; World Health Organization 2017). MDD contributes to around 800.000 suicide deaths annually (World Health Organization 2017), and after treatment, half of MDD patients relapse or have a recurrent episode within 2 years (Vittengl et al. 2007). Furthermore, the risk of recurrence is progressively increased with each successive episode (Solomon et al. 2000). This emphasizes the need for interventions to prevent relapses following MDD.

Increasing evidence points to cognitive deficits as an important part of MDD (Ahern et al. 2019; Ahern and Semkovska 2017; Hammar, Ronold, and Rekkedal 2022), and deficits are found in domains such as attention, executive functions (EF), and memory (McIntyre et al. 2013; Miskowiak et al. 2022). Cognitive deficits could predict worse treatment outcomes in MDD (Groves et al. 2018), and 79% of individuals with MDD report work functioning interference (Gilmour and Patten 2007; Lam et al. 2014). Furthermore, cognitive deficits are found to persist in remission and to worsen with each depressive episode (Hasselbalch et al. 2011; Semkovska et al. 2019). This could impact the quality of life (QoL) of individuals with a history of MDD.

MDD is associated with poorer QoL, even when symptoms are in remission (Angermeyer et al. 2002; IsHak et al. 2015, 2011). QoL is defined as: “an individual's perception of their position in life in the context of the culture and value systems in which they live and in relation to their goals, expectations, standards and concerns” (Whoqol Group 1995) (p. 1405). In a 10‐year follow‐up assessment, MDD patients still showed significantly poorer health‐related quality of life (HRQL) compared to healthy controls (Årdal et al. 2013). Physical, mental and social health can be measured objectively, and HRQL incorporates the perception leading to a subjective experience of one's health status (Testa and Simonson 1996). This subjective experience of health status will be applied in the current paper for defining HRQL. Several terms are often used synonymously with HRQL, such as “psychosocial function”, “social functioning”, “functioning” and “QoL” (Greer et al. 2010), and studies using these overlapping terms are cited in the current paper. QoL has been highlighted as a central outcome measure, and factors leading to reduced QoL in MDD should thus be identified and targeted (IsHak et al. 2011). Thus, studies investigating HRQL and how it relates to other residual symptoms following MDD are needed.

Cognitive deficits in MDD have been found to have a mediating effect on aspects of QoL such as functional outcomes and psychosocial impairment (McIntyre et al. 2013). Associations have been reported between cognitive deficits and psychosocial impairments, including poorer QoL, and poorer global, social, and occupational functioning in MDD samples (Cambridge et al. 2018). Mental HRQL, as assessed by the 36‐Item Short‐Form Health Survey (SF‐36), has been shown to be associated with cognitive deficits, especially EF, for patients with current MDD (Knight et al. 2020). EF can be understood as mechanisms that control and modulate lower‐level cognitive functions, such as working memory (WM), inhibition, and shifting (Miyake et al. 2000; Snyder et al. 2015). Individuals with both symptomatic and remitted MDD have reported that their cognitive deficits affected their QoL and psychosocial functioning (Ebert et al. 2017). There is most evidence for the mental, but not the physical aspects of HRQL, being associated with cognitive deficits (Knight et al. 2020; Shimizu et al. 2013). In sum, there is a growing body of evidence that cognitive deficits are associated with QoL‐related factors following MDD, which could increase the risk of relapse and recurrence.

EF shows the largest associations to psychosocial functioning in remitted MDD (Knight et al. 2018), and could thus influence QoL. Another study of remitted MDD found an association between delayed verbal recall and parts of HRQL (Shimizu et al. 2013). Cognitive deficits were found to be the main mediator of impaired occupational functioning in remitted MDD (Woo et al. 2016). In contrast, cognitive deficits were not associated with mental HRQL in a remitted MDD sample (Knight et al. 2020). Several authors advocate for the potential of cognitive remediation to enhance psychosocial functioning and QoL following depression (McIntyre et al. 2013; Knight et al. 2018; Woo et al. 2016). However, due to the sparse research on the association between QoL and cognitive deficits in remission from MDD, little is known about which aspects of cognition could be targeted for improving HRQL. Thus, it is important to employ broad measures when investigating these associations.

Objective (neuropsychological tests) and subjective measures of cognition (self‐report) show small associations in several studies (Mohn and Rund 2016; Srisurapanont et al. 2017; Wang et al. 2019). This could advocate for the utilization of both types of measures (Miskowiak et al. 2022; Serra‐Blasco et al. 2019). Subjective cognitive deficits are related to poorer QoL in MDD samples, even when adjusted for depressive symptom severity (Wang et al. 2019; Sumiyoshi et al. 2021; Zuckerman et al. 2018). Additionally, subjective deficits increase the probability of relapse (Wang et al. 2019; Sumiyoshi et al. 2021). Thus, both objective and subjective cognition could be relevant with regard to QoL. Despite cognitive deficits' impact on QoL, available MDD treatments do not sufficiently target these functionally relevant aspects (Zuckerman et al. 2018). Targeting cognitive functions could lead to increased QoL through functional recovery (Ahern et al. 2019; Buist‐Bouwman et al. 2008), and thus decrease relapse rates (Hoorelbeke et al. 2021). Recently, several interventions for improving cognition following MDD have been proposed.

Cognitive remediation (CR) is a term used for several behavioral interventions with the goal of improving cognitive performance through brain plasticity, i.e., structural changes in the brain caused by learning experiences (Johnston 2009). CR is usually defined as a combination of cognitive training (CT), transfer sessions, and/or psychoeducation (Bowie et al. 2013; Knight et al. 2021; Lee et al. 2013). The intervention in the current study is a narrower CT intervention. Cogmed was used in the current study (www.cogmed.com). This is a computerized working memory training (CWMT) program developed to improve WM functions. Several studies show benefits from CWMT (Brehmer et al. 2012; Klingberg et al. 2002; Spencer‐Smith and Klingberg 2015). CWMT delivered online is arguably more accessible and cost‐effective compared to interventions in more specialized clinical healthcare settings (Kueider et al. 2012). However, several aspects of CWMT, including long‐term and transfer effects to other domains like QoL, are not well established.

Effects of CT on daily functioning and QoL remain preliminary (Woolf et al. 2022). WM training is found to improve cognitive functions and could lead to neural changes by promoting brain plasticity (Pappa et al. 2020). However, the number of studies on MDD samples with HRQL‐related outcome measures is limited, and findings are inconclusive (Bowie et al. 2013; Knight et al. 2021; Lee et al. 2013; Naismith et al. 2011; Lengvenyte et al. 2020). Following WM training, effects have been found on tasks similar to training (near‐transfer), but whether this leads to changes in more distal domains (far‐transfer) remains debated (Pappa et al. 2020; Melby‐Lervåg et al. 2016). A systematic review found significant small effects of CR on daily functioning (Legemaat et al. 2021). Thus, the long‐term effect of CWMT on HQRL seems worth exploring.

There are several promising findings. A study using the same CWMT intervention as the current study found short‐term functional improvement, but no improvement in QoL measured by SF‐36 in a remitted sample with bipolar disorder (Lengvenyte et al. 2020). Previous investigations of the same sample as the current study found feasibility and acceptability of CWMT remitted depression. The main outcomes of the pilot study were drop‐out rates and goals completed during training, as well as improvement in WM and EF and rumination (Hammar, Semkovska, et al. 2022; Ronold et al. 2022). HRQL was also added as a secondary outcome. Since WM and EF are associated (McCabe et al. 2010), and several studies presented above highlight the importance of EF for psychosocial outcomes, it would be expected that improvements through CWMT could transfer to HRQL. Thus, the association between EF and HQOL, and long‐term improvements in HQOL following CWMT were assessed in the current study.

Several systematic reviews show improved cognitive function shortly following CR, but studies on long‐term effects are limited (Woolf et al. 2022; Goldberg et al. 2023) A longitudinal study found that subjectively measured cognitive deficits at baseline were associated with poorer QoL 6 months later (Sumiyoshi et al. 2021). This indicates the importance of long‐term assessment when investigating the effects of CR on HRQL. A 2‐year follow‐up study on the effectiveness of CR on subjective EF in MDD, as measured by the Behavior Rating Inventory of Executive Function for Adults (BRIEF‐A), found no significant improvements (Hagen and Stubberud 2021). In contrast, a longitudinal investigation found improvements on BRIEF‐A after 2 years (Ronold et al. 2023). A 1‐year follow‐up study on remitted MDD found improved objective cognition following CT, but no effects on daily life functioning were found (Hoorelbeke et al. 2021) Improved BRIEF‐A scores were also evident in an active control group. Thus, investigating associations between HRQL and cognitive functioning following MDD, as well as the long‐term effects of CWMT on HRQL, could improve the understanding of interventions in the remitted phase of the disorder.

This study aimed to investigate mental HRQL following CWMT in a remitted MDD sample, and associations between HRQL and EF. Finally, it was hypothesized that improvements in EF would be positively associated with improvement in mental HRQL. The research questions in the current study were the following:

1. Does mental HRQL improve following a CWMT intervention?
2. Is mental HRQL related to EF, and are improvements in EF related to improvement in HRQL?

## 2 Materials and Methods

### 2.1 Participants and Procedure

Participants were recruited from an outpatient clinic for affective disorders at Haukeland University Hospital, from previous research projects related to depression and through advertisements. Inclusion criteria were previous treatment for MDD and remission for the last 2 months as operationalized by a score of ≤ 12 on the Montgomery Åsberg Depression Rating Scale at baseline assessment (MADRS (Montgomery and Asberg 1979)). Exclusion criteria were self‐reported current alcohol or substance abuse, severe somatic illness, and earlier or current neurological disorder. A psychologist or psychiatric nurse confirmed the MDD diagnosis using the Norwegian version of the Mini‐International Neuropsychiatric Interview (Sheehan et al. 1998), and screened for exclusion criteria by a study questionnaire.

### 2.2 Participant Flow and Missing Data

Neuropsychological tests and self‐report measures were administered at T1, T2, T3, and T4. Assessments were performed at the neuropsychological clinic at the University of X by trained personnel. MADRS was missing for one participant at T1. The Conners' Continuous Performance Test‐II (CPT) Detectability was missing for one participant at T1. For SF‐36, one participant missed a question at T1, another participant missed a question at T3. The means for the relevant scale were computed and used as the item value. For one participant, all questions for the Role‐Emotional scale were missing at T3, resulting in 1‐year data for 11 participants at that scale.

The current study is a longitudinal pilot intervention study, with assessments pre‐intervention (T1), 5 weeks post‐intervention (T2), and follow‐up assessments after 1 year (T3) and 2 years (T4). It is a secondary analysis of data to investigate the effects of CWMT on HRQL, and how HRQL and EF are associated. The data were originally collected as part of a pilot study examining the feasibility and acceptability of CWMT for remitted MDD participants. The study was approved by The Regional Committee for Medical Research Ethics of X (X) and the X Data Inspectorate, and procedures were conducted in accordance with the Helsinki Declaration. Informed written consent was provided by all participants, and they were compensated with 400 Norwegian Kroner (approximately 40 USD) and free access to the CWMT program.

### 2.3 CWMT Intervention

Cogmed was used in the current study. The participants underwent 25 online sessions of CWMT for 5 weeks; each session contained various symbol and spatial sequence WM tasks and lasted 30–40 min. All parts of the intervention occurred individually, and the program increased in difficulty based on the participants' performance level. Three times throughout the training, participants chose personal goals and answers to motivational questions in the program and had weekly telephone contact with a trained coach for feedback and motivation.

### 2.4 Self‐Report Measures

#### 2.4.1 SF‐36

The Norwegian version of SF‐36 was used to assess HRQL (Loge et al. 1998; Ware and Sherbourne 1992). SF‐36 was constructed for the Medical Outcomes Study and consists of eight health concepts. Factor analysis revealed two clusters of health concepts, labeled mental and physical (Ware 1995), and the four mental health scales Vitality, Social Functioning, Mental Health, and role limitations due to emotional problems (Role Emotional) were used in the current study. Scores range from 0 to 100, where lower scores indicate more disability. The Norwegian version of SF‐36 has been found to have high construct validity and psychometric properties (Loge et al. 1998; Garratt and Stavem 2017).

#### 2.4.2 BRIEF‐A

The Norwegian version of the 75‐item self‐report version of BRIEF‐A measured subjective EF (Nicholas and Solbakk 2006). Higher scores indicate poorer self‐perceived EF (Roth et al. 2005). BRIEF‐A measures the individual's self‐perceived everyday functioning related to EF. The Metacognition Index (MI), the Behavior Regulation Index (BRI), and the Global Executive Composite score (GEC) were used as measures of subjective EF. MI is based on the clinical scales Initiate, Working Memory, Plan/Organize, Task Monitor, and Organization of Materials, and BRI is based on the four clinical scales Inhibit, Shift, Emotional Control, and Self‐Monitor. GEC is composed of both indexes.

### 2.5 Objective Measures of EF

Tests from the Delis‐Kaplan Executive Functioning System (Delis et al. 2001) were included in the study to measure the EF switching and inhibition (Color‐Word Interference test and trail making test), attentional control, and inhibition was measured by CPT‐II (Conners 2002), and WM was measured by digit span and IQ estimated by the first edition of the Wechsler abbreviated test of intelligence (Wechsler 1997). For further information about the tests, see Appendix.

### 2.6 Statistical Methods

All statistical analyses were conducted using the Statistical Package for the Social Sciences (Version 29.0 for Windows). The SF‐36 scoring system was used to recode raw scores to a 0–100 scale (Ware 2000). The significance level was set at *p* = 0.05, and a one‐tailed significance level was used to investigate the predicted HRQL improvement, as well as the expected positive correlations between CWMT improvement and improved HRQL change scores. Effect sizes (Pearson's *r* and Cohen's *d*) were interpreted as small if *d* = 0.2, moderate if *d* = 0.5, or large if *d* = 0.8 (Cohen 1988). In addition, *z*‐scores were conducted for the SF‐36 scales to compare sample means with the Norwegian normative data (Garratt and Stavem 2017) for males and females aged 30–39.

Scatterplots and histograms were used to screen for outliers in the data. Parametric tests were used for all analyses, after assessing normality by the Kolmogorov–Smirnov test. Independent samples *t*‐tests were used to compare completers of T2 and T4 with non‐completers, assessing for potential attrition effects. Because of drop out and low power to detect significant changes, several paired sample *t*‐tests were used over multivariate ANOVA to investigate the development of HRQOL after 5 weeks, 1‐ and 2‐years following the CWMT intervention. To assess the relationship between HRQOL and EF, the bivariate correlation coefficient (Pearson's *r*) was used. Pearson's *r* was also used to assess the correlation between the change scores from T1 to T4 of EF measures that significantly correlated with SF‐36. Change scores for BRIEF‐A MI were calculated T1–T4 and T4–T1 for SF‐36, so that positive values indicated improvement. *T*‐scores were used for CPT Detectability, and raw scores were used for all other objective EF measures.

## 3 Results

### 3.1 Baseline Characteristics and Attrition Analysis

Descriptive statistics conducted with the 29 participants showed a mean age of 36.21.

(SD = 10.89), an average of 16.21 years of education (SD = 1.85), a mean WASI score of.

115.82 (SD = 8.06) indicating a normal level of intellectual functioning, and a MADRS score of 6.50 (SD = 6.21) indicating that the sample was remitted. Ten participants were male. Demographic characteristics, neuropsychological, and self‐report measures for completers and non‐completers are presented in Table 1. Attrition analyses showed no significant differences between completers and non‐completers on any variables. Standardized scores for BRIEF‐A and SF‐36 (Garratt and Stavem 2017; Roth et al. 2005) are presented in Table 2. 62% and 31% of participants scored the maximum score of 100 for Role‐Emotional and Social Functioning, respectively. For Role‐Emotional, 20% of participants scored the minimum score of 0.

**TABLE 1:** Means and SD at baseline for T2 and T4 completers, and T2 and T4 non‐completers^a^.

Baseline characteristics	*M* (SD)			
	T2 non‐completers *n* = 9	T2 completers *n* = 20	T4 non‐completers *n* = 19	T4 completers *n* = 10
Age	33.56 (7.73)	37.40 (12.04)	36.05 (11.43)	36.50 (10.38)
Years of education	15.44 (1.74)	16.55 (1.85)	16.32 (1.77)	16.00 (2.11)
MADRS^b^	5.50 (3.12)	6.70 (6.71)	7.14 (5.70)	5.60 (7.90)
IQ	116.56 (7.63)	115.50 (8.43)	117.16 (8.86)	113.30 (5.89)
SF‐36				
Vitality	42.8 (17.9)	51.8 (15.7)	46.3 (17.9)	54.00 (13.3)
Mental health	67.6 (15.5)	70.8 (8.9)	67.8 (11.7)	73.6 (9.5)
Social functioning	72.2 (20.5)	74.4 (23.5)	70.4 (23.3)	80.00 (19.7)
Role‐emotional	63.0 (45.5)	73.3 (41.3)	64.9 (45.1)	80.00 (35.8)

*Note:* T2 completers were compared to T2 non‐completers, and T4 completers to T4 non‐completers. No significant differences were found in any variable.

Abbreviations: BRI, Behavior Regulation Index; BRIEF‐A, Behavior Rating Inventory of Executive Functions‐Adult; GEC, Global Executive Composite; IQ, intelligence quotient; MADRS, Montgomery Åsberg Depression Rating Scale; MI, Metacognition Index; SF‐36, 36‐Item Short‐Form Health‐Survey; WASI IQ, Wechsler's Abbreviated Scale of Intelligence Quotient.

^a^ T2 completers and T4 completers were those who completed assessment respectively after 5 weeks (T2) and 2 years (T4). T2 non‐completers and T4 non‐completers dropped out respectively before assessment at T2 and T4.

^b^ MADRS: *N* = 4 for T2 non‐completers, and *n* = 14 for T4 non‐completers.

**TABLE 2:** SF‐36 Z‐scores and BRIEF‐A T‐scores at baseline.

Variable	*z‐* or *t‐*scores, *N* = 29
SF‐36^a^	
Vitality	*z* = −0.58
Mental health	*z* = −0.67
Social functioning	*z* = −0.76
Role‐emotional	*z* = −0.68
BRIEF‐A^b^	
MI	*t* = 61.7 (13)
BRI	*t* = 52.9 (8.9)
GEC	*t* = 58.6 (10.9)

^a^ SF‐36 *z*‐scores compare sample means to Norwegian norms (Garratt and Stavem 2017) for males and females aged 30–39.

^b^ BRIEF‐A *t*‐scores are presented as follows: *M* (SD).

### 3.2 HRQL Following CWMT

Results showed non‐significant changes in HRQL at T2 and T3; however, a significant moderate improvement was apparent on the SF‐36 Mental Health scale 2 years after CWMT. Results are presented in Table 3.

**TABLE 3:** Changes in SF‐36 at T2, T3, and T4, all compared to T1.

Variable	*M* (SD)	*t*	df	One‐sided *p*	Cohen's *d*
Vitality					
T1, *N* = 29	48.9 (16.6)				
T2, *n* = 20	49.0 (18.7)	−0.71	19	0.244	−0.16
T3, *n* = 12	48.8 (21.9)	−1.14	11	0.139	−0.33
T4, *n* = 10	60.0 (17.8)	1.04	9	0.163	0.33
Mental health					
T1, *N* = 29	69.7 (11.1)				
T2, *n* = 20	70.8 (15.2)	0.00	19	0.500	0.00
T3, *n* = 12	69.3 (13.4)	−1.05	11	0.159	−0.30
T4, *n* = 10	79.6 (9.5)	2.09	9	0.033*	0.66
Social functioning					
T1, *N* = 29	73.7 (22.2)				
T2, *n* = 20	74.4 (23.1)	0.00	19	0.500	0.00
T3, *n* = 12	76.0 (30.4)	0.51	11	0.311	0.15
T4, *n* = 10	88.8 (13.8)	1.17	9	0.136	0.37
Role‐emotional					
T1, *N* = 29	70.1 (42.1)				
T2, *n* = 20	74.4 (23.1)	−0.19	19	0.427	−0.04
T3, *n* = 11	69.7 (40.7)	−0.86	10	0.205	−0.26
T4, *n* = 10	83.3 (32.4)	0.36	9	0.363	0.11

* One‐tailed *p* < 0.05.

### 3.3 Associations Between HRQL and EF

The BRIEF‐A indexes MI and GEC showed significant moderate negative correlations to SF‐36 scales measuring Mental Health and Role‐Emotional. There were only small, nonsignificant associations between HRQL and neuropsychological tests. See Table 4 for more information about associations between the SF‐36 and objective and subjective EF measures at T1.

**TABLE 4:** Correlations between SF‐36, BRIEF‐A, and objective EF measures at baseline, *N* = 29.

Variable	1	2	3	4	5	6	7	8	9	10	11	12
SF‐36												
1. Mental Health	1											
2. Social Functioning	0.35	1										
3. Role‐Emotional	0.54**	0.26	1									
4. Vitality	0.43*	−0.02	0.43*	1								
BRIEF‐A												
5. BRI	−0.33	−0.13	−0.31	−0.02	1							
6. MI	−0.47*	−0.19	−0.65**	−0.28	0.57**	1						
7. GEC	−0.47**	−0.19	−0.60**	−0.22	0.79**	0.96**	1					
8. WAIS Digit Span	−0.04	0.15	−0.20	−0.07	−0.06	0.20	0.13	1				
9. TMT Switching	0.13	−0.16	0.07	0.22	0.09	−0.04	0.00	−0.19	1			
CWIT												
10. Inhibition	0.00	−0.12	0.14	−0.03	0.07	0.10	0.10	−0.41*	0.13	1		
11. Inhibition/switching	0.24	0.16	0.24	0.17	−0.01	−0.08	−0.06	−0.09	0.31	0.39*	1	
12. CPT detectability^a^	−0.05	−0.01	−0.07	0.24	0.19	0.12	0.16	−0.19	0.06	0.44*	0.24	1

*Note:* Raw scores were used for every variable except for CPT Detectability (*t*‐scores).

^a^ *n* = 28 for CPT detectability.

* Two‐tailed *p* < 0.05.

** Two‐tailed *p* < 0.01.

Due to significant correlations between MI and Mental Health and Role Emotional at baseline, as well as a significant improvement in Mental Health at T4, correlations between CWMT improvement, change in Mental Health, and change in MI from T1 to T4 were explored (See Table 5). No significant correlation was found between improved HRQL and improved subjective EF. However, CWMT improvement showed a small to moderate association to MI change, and SF‐36 Mental Health and Role Emotional (*p* = 0.099, *p* = 0.074, *p* = 0.104, respectively).

**TABLE 5:** Correlations between CWMT improvement and SF‐36 and BRIEF‐A T1‐T4 change, *N* = 10.

Variable	1	2	3	4
1. CWMT improvement	1			
2. BRIEF‐A MI	0.445	1		
SF‐36 Mental Health	0.492	−0.01	1	
SF‐36 Role Emotional	0.419	0.291	0.195	1

Abbreviation: MI, meta cognition index raw change score.

## 4 Discussion

The current study aimed to investigate how mental HRQL developed following CWMT and its relationship with EF in remission from MDD. The results indicated moderate improvements in HRQL Mental Health after 2 years. In addition, a significant moderate negative correlation between subjective EF deficits and HRQL was evident at baseline.

### 4.1 HRQL Improvement Following CWMT Intervention

Significant improvements in HRQL were evident, but only after 2 years. Thus, there was partial support for research question 1. The results did not show improvement in HRQL from T1 to T2 or T3. The lack of short‐term effects is similar to findings in a bipolar group using similar methods (Lengvenyte et al. 2020). Results might have been different for a sample with a lower baseline HRQL. E.g. improved scores at T2 were not possible for a considerable portion of the sample on Role‐Emotional due to ceiling effects. Regression to the mean could occur for participants with high scores (Barnett et al. 2005; Duff 2012; Stigler 1997). Alternatively, the time interval of approximately 5 weeks was too short to see transfer effects on HRQL, and longer follow‐up might be necessary for these improvements to manifest. In sum, methodological issues may have contributed to the lack of short‐term improvements in HRQL.

Results showed significant moderate improvement on the SF‐36 Mental Health scale after 2 years. These findings are promising but should be considered preliminary. The study is small and the data is collected as part of a pilot study without a control group or blinding. Findings could support that the time interval between the intervention and T2 may have been too short for neural and cognitive improvement to transfer to behavioral changes. In research on CR for schizophrenia, a lack of QoL improvement has similarly been explained by short time intervals (d'Amato et al. 2011), and adequate time has been argued to be necessary for sufficient opportunities to practice new skills to see a transfer from cognitive enhancement to everyday functional outcomes (Pappa et al. 2020; Bowie et al. 2012). However, findings are not fully explained by the time interval, considering the lack of HRQL improvements after 1 year. Improvements in EF have been found in two longitudinal studies on CT in remitted MDD (Hoorelbeke et al. 2021; Ronold et al. 2023), while one study did not find improvements in a mixed MDD sample (Hagen and Stubberud 2021). The current study adds to a preliminary trend of long‐term effects of CT in remitted MDD with improvements in HRQL Mental Health and underscores the need for more long‐term follow‐up studies of QoL following CT.

Notably, the study design cannot establish the CWMT intervention as causal for improved HRQL; however, the small associations between CWMT and HRQL discussed below could suggest this. The notion that improving cognition would improve QoL is plausible considering findings of cognition as a mediator of QoL in MDD (McIntyre et al. 2013), associations between EF deficits and SF‐36 assessed mental HRQL (Knight et al. 2020), and associations between EF deficits and psychosocial functioning in remitted MDD previously reported in the literature (Knight et al. 2018). This should be pursued by future longitudinal studies.

### 4.2 Associations Between HRQL and EF

There were significant associations between HRQL and subjective measures of EF. These measures may better reflect real‐world behaviors and performance compared to the structured context of objective neuropsychological assessment (Snyder et al. 2021), and thus be more related to daily activities like occupational functioning (Barkley and Fischer 2011), which are proposed to be more closely related to HRQL. Findings are in line with studies showing small associations between objective and subjective EF and might reflect that they measure largely independent constructs (Snyder et al. 2021; Friedman and Gustavson 2022). Also, SF‐36 and BRIEF‐A are both self‐reported questionnaires, which in general are found to be more highly correlated (Dang et al. 2020). The correlations between BRIEF‐A indexes MI and HRQL suggest that poorer subjective EF is associated with lower Mental Health and Role Emotional. Similar correlations between QoL and BRIEF‐A have been reported (Sharfi and Rosenblum 2016; Zhang et al. 2021), supporting the results of the current study and that BRIEF‐A measures aspects that are perceived as important for daily life. The slightly higher MI than BRI at baseline could indicate more deficits in metacognition. In another remitted sample, MI scales also showed the strongest associations with the other residual symptoms (Hoorelbeke et al. 2022), suggesting that MI deficits initiate dysfunctional cognitive and depressive patterns, and that perceived cognitive deficits are more detrimental to function than emotional regulation difficulties measured by the BRIEF. This highlights the clinical MI in future studies of remitted MDD and illustrates the association between HRQL and subjective EF deficits in metacognition.

### 4.3 Associations Between Improvement in EF and HRQL

There were no significant correlations between EF improvement and HRQL. Small to moderate correlations between CWMT improvement and MI and HRQL were found, suggesting effects from the intervention. However, this remains speculative with the current design. Only small correlations between EF and HRQL improvement were found. Interestingly, more association between Role emotional and MI change than change in mental health could hint at a mediating role of improved daily life functioning for Mental Health improvement that could be pursued by future studies. Individualization might also be relevant for optimal effects on HRQL.

One study showing improvements in psychosocial functioning had an intervention that was adapted to participants' cognitive deficits (Lee et al. 2013). Studies that find improved psychosocial functioning have had broader interventions, such as training of multiple cognitive domains (Knight et al. 2021), psychoeducation on cognitive deficits (Lee et al. 2013), and focus on hot cognition by training social‐cognitive and emotional abilities in addition to cold cognition (Knight et al. 2021). It is nonetheless worth noting that the CR studies that found improved psychosocial functioning differ from the current study on several factors, such as samples of first episode MDD and psychosis (Lee et al. 2013), a combination of remitted and current MDD (Knight et al. 2021), and treatment‐resistant depression (Bowie et al. 2013). These findings indicate that elements such as broader and individualized CT, in addition to psychoeducation and transfer sessions, could be relevant factors to see the optimal effect of CT on HRQL.

### 4.4 Strengths, Limitations and Future Directions

A limited number of studies have examined the effects of CWMT on domains of daily functioning (Woolf et al. 2022), and 2‐year follow‐up data can also be considered a major strength. A comprehensive number of measures pre‐ and post‐intervention were conducted. Additionally, utilizing the four mental subscales of SF‐36 in our analysis may have given more accurate and detailed results (Taft et al. 2001).

The lack of a control group precludes isolating the effect of CWMT on HRQL, and the observed change in HRQL could be due to demand characteristics and/or random variation. Placebo effects in CT have previously been demonstrated (Foroughi et al. 2016), but this seems unlikely given that HRQL improvement was not found acutely or 1 year after the intervention. Due to small sample size, Bonferroni corrections were not conducted to reduce the risk of type‐II errors (Nakagawa 2004), which augments the possibility of type‐I errors. Future studies should employ more robust statistical methods that correct for multiple comparisons, and the findings in the current study should be considered preliminary before they are replicated in larger, more controlled studies. The small sample size and drop out might also have led the current study to be underpowered to detect other changes. Analysis did not indicate significant attrition effects; however, certain methodological issues with SF‐36 are worth noting (Rose et al. 1999; Torrance et al. 2009), and a discussion of these can be found in the Appendix.

Additional studies are needed to investigate the long‐term effects of CWMT on functional outcomes such as HRQL, with adequately sampled randomized controlled trials. For future randomized controlled studies on this matter, the inclusion of participants with low baseline QoL measures, subjective measures of EF, and sufficient follow‐up time is suggested by the current results and a recent systematic review on cognitive remediation, indicating that sufficient follow‐up time is needed for effects in daily function measures (Vita et al. 2024), like HRQL. Furthermore, studies should investigate whether additional aspects of cognitive functioning than WM should be targeted and if interventions should be personalized for HRQL to improve optimally. Finally, the effect of other intervention elements such as psychoeducation and transfer elements on QoL should be investigated further, and recent best practice guidelines suggest that more comprehensive cognitive remediation interventions than merely WM training result in the largest effects (Wykes et al. 2024) Personalized, comprehensive cognitive training interventions with adequate follow‐up times should be pursued in well‐designed studies to establish effects on HRQL and relapse prevention.

## 5 Conclusions

The current study found significantly improved quality of life for remitted MDD participants 2 years after a CWMT intervention. At baseline, quality of life aspects like mental health correlated with subjectively rated meta cognition, but not with objective EF measures. The study gives preliminary support for CWMT as a possible cost‐effective method of increasing QoL and thus potentially preventing recurrent episodes in remitted MDD. In conclusion, results could indicate long‐term effects of CWMT on HRQL in remitted MDD, but further studies with larger samples and more designs are needed to establish these effects.

## Author Contributions

**Eivind Haga Ronold:** conceptualization, methodology, statistical analysis, supervision, writing – review and editing. **Silje Klundelien Storfossen:** writing – original draft, review and editing. **Marta Vikingstad Vignes:** writing – original draft, review and editing. **Åsa Hammar:** conceptualization, resources, investigation, methodology, writing – review and editing, supervision.

## Funding

This work was supported by Universitetet i Bergen.

## Conflicts of Interest

E.H.R. has taught a seminar on the Wechsler Adult Intelligence Scale for Pearson, who is the commercial owner of the intervention used in the current study. E.H.R. is also contributing to the development of the new Scandinavian version of the Wechsler Adult Intelligence Scale and will receive an honorarium for this. E.H.R. has no financial relationship with Pearson concerning the intervention used in the current study, and the current study was conducted before the relationships above were initiated and is unrelated to the current study. S.K.S., M.V.V., and Å.H. have no disclosures.

## Supporting information


**Appendix:** sjop70060‐sup‐0001‐Appendix.pdf.

## Data Availability

The data that support the findings of this study are available on request from the corresponding author. The data are not publicly available due to privacy or ethical restrictions.
