# Supplementary material for: A Secondary Analysis of Longitudinal Pilot‐Study Data Investigating the Associations Between Health‐Related Quality of Life and Executive Functions in Remitted Major Depressive Disorder and Developments Two‐Years Following Cognitive Training
Source: Scand J Psychol. 2025 Dec 14;67(3):665–75. doi: 10.1111/sjop.70060 (PMC13159509; doi:10.1111/sjop.70060)
Supplement: Supplementary file 1 — Appendix: sjop70060‐sup‐0001‐Appendix.pdf. [file SJOP-67-665-s001.pdf]

Objective neuropsycological tests

D-KEFS Color-Word Interference Test-3 (CWIT-3): CWIT is based on the Stroop paradigm and is a D-KEFS subtest (Delis et al., 2001), a commonly used test battery for assessment of EF. In the Inhibition condition, the participant is presented with the words “blue”, “green” and “red” printed incongruently in blue, green or red ink. The participant is then asked to not read the words but report the ink color as quickly as they can. The condition is thought to measure overlearned response inhibition. Performance is measured by completion time, and lower scores indicate better performance.

D-KEFS CWIT-4: In this condition the participant is presented with the words “blue”, “green” and “red” written in blue, green or red ink, and some of the words are enclosed in boxes. The participant is asked to report the ink color of the word and not read it except when the word is enclosed in a box, then the participant is asked to read the word aloud and ignore the ink color. This condition measures inhibition and switching. As with the Inhibition condition, lower scores indicate better performance.

D-KEFS Trail Making Test-4 (TMT-4). TMT Switching is a D-KEFS subtest (Delis et al., 2001) and is conducted with paper and pencil. The condition measures switching, an aspect of EF. The participant must draw a line as fast as possible, while switching between finding the next number in a rising order, and the next letter in an alphabetical order. Low completion time indicates better performance.

CPT-II Detectability

Further aspects of impulsivity was measured by CPT-II (Conners, 2002) the participant is instructed to look at a computer screen where letters appear with intervals of 1, 2, or 4 seconds. The participant must avoid pressing a key when an «X» appears, while pressing the key when any other letter appears. Commission errors, failing to press a key when supposed to, is a measure of inattentiveness. Omission errors, pressing the key when supposed to avoid it, measures inhibition. CPT Detectability is based on the participant’s ability to distinguish non-targets from targets and is a measure of omission and commission errors. Lower scores indicate better performance.

Wechsler Adult Intelligence Scale-III (WAIS-III) Digit Span

Auditory working memory, which can be considered an aspect of EF, was measured by Digit Span from the WAIS-III (Wechsler, 1997). In the forward condition, a number sequence is read to the participant, who is asked to repeat the sequence. One number is added to the sequence length every other trial, so the level of difficulty gets progressively higher. In the backward condition the participant must repeat the sequence backwards, requiring holding and manipulating information in working memory. The Digit Span score is calculated based on performance on both the forward and backward condition. Higher scores indicate better performance.

Wechsler Abbreviated test of intelligence (WASI)

WASI estimates intelligence scores from two verbal- and two visual-ability tests from WAIS-III (Wechsler, 1997).

Statistical methods and scoring

All statistical analyses were conducted using the Statistical Package for the Social Sciences (Version 29.0 for Windows). The SF-36 scoring system was used to recode raw scores to a 0-100 scale (Ware, 2000). Significance level was set at $p = 0.05$, and a one-tailed significance level was used the predicted direction of HRQOL development, as well as correlations between CWMT improvement and change scores.

Independent samples t-tests were used assess for potential attrition effects. Paired sample t-tests were used to investigate development of HRQOL. Pearson´s r was used to assess the correlations between CWMT improvement, HRQOL and EF, and correlations between the CWMT improvement and change scores from T1 to T4 for BRIEF-A MI and SF-36 Mental health and Role Emotional. T-scores were used for CPT Detectability, and raw scores were used for all other objective EF measures. .

Percentage of participants with minimum and maximum scores score on the SF-36 scales were calculated. Z-scores were calculated for the SF-36 scales to compare sample means with the Norwegian normative data (Garratt & Stavem, 2017). Percent improvement on CWMT was calculated by subtracting starting score from finishing score and dividing by 100. Change scores for BRIEF-A and SF-36 was calculated by subtracting T1 from T4 scores so that positive values represented improvements.
